# *De novo* genome assembly of the marine teleost, bluefin trevally (*Caranx melampygus*)

**DOI:** 10.1093/g3journal/jkab229

**Published:** 2021-07-14

**Authors:** Brandon D Pickett, Jessica R Glass, Perry G Ridge, John S K Kauwe

**Affiliations:** 1 Department of Biology, Brigham Young University, Provo, UT 84602, USA; 2 South African Institute for Aquatic Biodiversity, Makhanda 6139, South Africa; 3 College of Fisheries and Ocean Sciences, University of Alaska Fairbanks, Fairbanks, AK 99775, USA; 4 University President’s Office, Brigham Young University—Hawai‘i, Laie, HI 96762, U SA

**Keywords:** bluefin trevally, ‘Omilu, Carangidae, *de novo* genome assembly, MSMC

## Abstract

The bluefin trevally, *Caranx melampygus*, also known as the bluefin kingfish or bluefin jack, is known for its remarkable, bright-blue fins. This marine teleost is a widely prized sportfish, but few resources have been devoted to the genomics and conservation of this species because it is not targeted by large-scale commercial fisheries. Population declines from recreational and artisanal overfishing have been observed in Hawai‘i, USA, resulting in both an interest in aquaculture and concerns about the long-term conservation of this species. Most research to-date has been performed in Hawai‘i, raising questions about the status of bluefin trevally populations across its Indo-Pacific range. Genomic resources allow for expanded research on stock status, genetic diversity, and population demography. We present a high quality, 711 Mb nuclear genome assembly of a Hawaiian bluefin trevally from noisy long-reads with a contig NG50 of 1.2 Mb and longest contig length of 8.9 Mb. As measured by single-copy orthologs, the assembly was 95% complete, and the genome is comprised of 16.9% repetitive elements. The assembly was annotated with 33.1 K protein-coding genes, 71.4% of which were assigned putative functions, using RNA-seq data from eight tissues from the same individual. This is the first whole-genome assembly published for the carangoid genus *Caranx*. Using this assembled genome, a multiple sequentially Markovian coalescent model was implemented to assess population demography. Estimates of effective population size suggest population expansion has occurred since the Late Pleistocene. This genome will be a valuable resource for comparative phylogenomic studies of carangoid fishes and will help elucidate demographic history and delineate stock structure for bluefin trevally populations throughout the Indo-Pacific.

## Introduction

**Figure 1:**
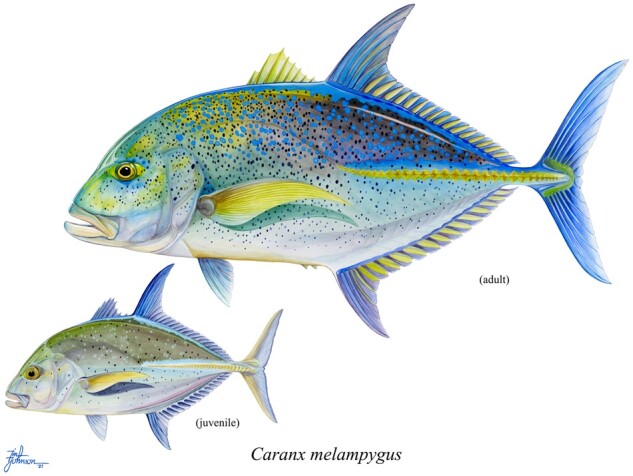
Bluefin trevally (*C. melampygus*) adult and juvenile. Quantitative morphological data for this illustration of *C. melampygus* were obtained primarily from (Heemstra *et al.* 2021). These were then evaluated by the artist who selected specific values for details such as number of lateral line scutes (32), number of rays (23), and spines (8) in the dorsal fin, and number of rays (19) and spines (2) in the anal fin. Each of these was portrayed in the illustration to be near the middle of the ranges reported. Illustration copyright: Tim Johnson, used with permission.

The bluefin trevally (*Caranx melampygus*; Cuvier 1833) is a marine teleost fish (Carangiformes: Carangoidei) inhabiting coastal environments throughout the tropical and subtropical Indo-Pacific (Figure 1). *C. melampygus* is a top predator on coral and rocky reef ecosystems, reaching up to 117 cm in length and feeding predominantly on shallow-water fishes and invertebrates (Sudekum *et al.* 1991; Meyer *et al.* 2001). In the Northwestern Hawaiian Islands, for example, bluefin trevallies consume an estimated 11,000 metric tons of prey per year, confirming their role as important predators in this region (Sudekum *et al.* 1991). *C. melampygus* is also targeted by small-scale and recreational fisheries in Hawai‘i, where it is known by its Native Hawaiian name, ‘*omilu* (Meyer *et al.* 2001). In recent decades, the *C. melampygus* population in Hawai‘i has been impacted by overharvesting and habitat destruction (Friedlander and Dalzell 2004). For this reason, there has been significant interest in Hawai‘i in captive breeding for aquaculture (Moriwake *et al.* 2001; Zhao and Lu 2006). Because the bulk of research on the bluefin trevally has been conducted in Hawai‘i, observations of population declines raise concerns for populations in other parts of its range, where abundance and biomass estimates remain unknown.

Recent genomic evidence suggests *C. melampygus* comprises a unique population in Hawai‘i compared with several localities sampled across the Indo-Pacific (Glass *et al.* 2021), and an analysis of complete mitochondrial genomes suggests individuals from Guam are also genetically distinct (Genomic Resources Development Consortium et al. 2014). Given population declines and evidence of unique stock structure in Hawai‘i, whole-genome data for *C. melampygus* would provide unprecedented value for inferring demographic history, estimating effective population size, and testing for selection and local adaptation. Juvenile and adult individuals frequently utilize estuarine habitats, for example, and have a strong tolerance for freshwater in coastal locations where estuaries are present (Blaber and Cyrus 1983). Studying the evolution and physiology of *C. melampygus* in a genomic context is valuable to the broader scientific and reef fish community, especially given interest in the genomic mechanisms of adaptation of marine and anadromous fishes to freshwater (Kültz 2015). Furthermore, whole-genome data provide baseline biological information for delineating wild stocks, a critical component of transboundary fisheries management, while also serving as an important reference for the aquaculture industry to examine genomic signatures of growth in captivity and susceptibility to disease (Zhao and Lu 2006). At present, published whole-genome data are available for only 7 out of ∼150 carangoid species: *Echeneis naucrates* (Linnaeus 1758; Koepfli *et al.* 2015), *Trachinotus ovatus* (Linnaeus 1758; Zhang *et al.* 2019), *Selene dorsalis* (Gill 1863; Malmstrøm *et al.* 2017), and four *Seriola* sp. (Purcell *et al.* 2015; Ozaki and Araki 2017; Araki *et al.* 2018; Yasuike *et al.* 2018), all of which diverged from *C. melampygus* ∼48–50 Mya (Harrington *et al.* 2016). Here, we present an annotated *de novo* genome assembly of *C. melampygus* to facilitate future research for aquaculture development and expand the genomic resources of carangoid fishes for comparative phylogenomic analysis.

## Materials and methods

An overview of the methods used in this study is provided here. Where appropriate, additional details, such as the code for custom scripts and the commands used to run software, are provided in the Supplementary Bioinformatics Methods (Supplementary File S1).

### Sample acquisition and sequencing

One *C. melampygus* individual was captured in 3–9 m of water <1 km off the coast of O‘ahu (near Kaneohe, Hawai‘i, USA: 21° 26′ 45.3″ N 157° 48′ 07.5″ W) in April 2018. The specimen was caught using a Shimano (Sakai, Osaka, Japan) ocean rod outfitted with a Daiwa (Cypress, CA, USA) Saltiga 6500 reel and a white feather jig. Brain, eye, fin, gill, heart, kidney, liver, and muscle tissue samples were collected immediately upon capture, flash-frozen in liquid nitrogen, and packaged in dry ice for transportation to Brigham Young University (BYU; Provo, UT, USA) for storage at −80° until sequencing. All tissue samples were used for short-read RNA sequencing. The heart tissue was also used for long-read DNA sequencing.

High-molecular weight (HMW) DNA was extracted and prepared for long-read sequencing following the protocol “Procedure & Checklist—Preparing >30 kb SMRTbell Libraries Using Megaruptor Shearing and BluePippin Size-Selection for PacBio RS II and Sequel Systems.” Briefly, HMW DNA was extracted using a wide-bore pipette tip and quality was assessed using an Agilent (Santa Clara, CA, USA; https://www.agilent.com) Fragment Analyzer and Sage Science (Beverly, Massachusetts, USA; https://sagescience.com) Pippin Pulse. DNA quantitation was performed at each step using a Beckman Coulter (BC; Brea, CA, USA; https://www.beckman.com) Qubit v3.0 Fluorometer. No shearing, for example, with a Diagenode (Seraing, Belgium; https://www.diagenode.com) Megarupter, was performed because the DNA was already sufficiently fragmented (average size of 25 Kb). After exonuclease removal and damage and end repairs, adapter ligation was performed using a Pacific Biosciences (PacBio; Menlo Park, CA, USA; https://www.pacb.com) SMRTbell Library Kit. Purification was performed using BC AMPure PB beads, and the resulting DNA was size selected for fragments >20 Kb with the Sage Science BluePippin. Continuous long-read (CLR) sequencing was performed on ten SMRT cells for a 10-h movie on the PacBio Sequel at the BYU DNA Sequencing Center (DNASC; https://dnasc.byu.edu), a PacBio Certified Service Provider.

RNA was extracted using Invitrogen (part of Thermo Fisher Scientific, Carlsbad, CA, USA; https://www.thermofisher.com) TRIzol Reagent (Catalog Numbers 15596018 and 15596026), as described in the User Guide (Document Part Number 15596026.PPS; Publication Number MAN0001271, Revision B.0). The quality and quantity of the RNA were assessed using an Agilent Fragment Analyzer with their RNA standard sensitivity kit (Part Number DNF-471-0500), and 4 µg of RNA was prepared for sequencing with Roche (Basel, Switzerland; https://sequencing.roche.com) KAPA Stranded RNA-Seq kit, following recommended protocols as described in the Technical Data Sheet titled “KAPA Stranded mRNA-Seq Kit Illumina platform” with product code KR0960, version 3.15 (this is now outdated, and newer versions can be found on Roche’s website at https://sequencing.roche.com/en-us/products-solutions/by-category/library-preparation/rna-library-preparation/kapa-stranded-rna-seq-kits/ordering.html). The average insert size of the library was 300 bp. Paired-end RNA sequencing was performed in Rapid Run mode for 250 cycles with the eight samples across two lanes on the Illumina (San Diego, CA, USA; https://www.illumina.com) Hi-Seq 2500 at the DNASC.

### Sequence assembly

The PacBio CLR reads were self-corrected and assembled with Canu v1.6 (Koren *et al.* 2017). Repeat characterization was performed with RepeatMasker v4.1.2-p1 (Smit *et al.* 2021) using Dfam v3.3 (Storer *et al.* 2021) and the RepBase RepeatMasker Library v20181026 (Jurka 1998; Bao *et al.* 2015). Assembly continuity statistics, for example, N50 and area under the NG-curve (auNG; Li 2020), were calculated with caln50 downloaded April 2020 (https://github.com/lh3/calN50) and a custom Python (https://www.python.org) script. The genome size provided to Canu and used for the computation of assembly statistics was based on values recorded in the Animal Genome Size Database (Gregory 2018). A C-value was not listed in the database for *C. melampygus*; we used 0.8 (782.4 Mb) as an upper limit based on recorded genome size values for other *Caranx* species.

The transcriptome was assembled from Illumina RNA-seq reads from all eight tissues (*i.e.*, brain, eye, fin, gill, heart, kidney, liver, and muscle). The RNA-seq reads were not corrected, but the quality was assessed using fastqc (Babraham Bioinformatics Group 2015). The transcripts were assembled using Trinity v2.6.6 (Grabherr *et al.* 2011). Both the genome and transcriptome assemblies were assessed for completeness using single-copy orthologs with BUSCO v4.0.6 (Simão *et al.* 2015) and the Actinopterygii subset of OrthoDB v10 (Kriventseva *et al.* 2019).

### Computational genome annotation

The MAKER v3.01.02-beta (Holt and Yandell 2011) pipeline was used to annotate the genome assembly. Generally speaking, annotation proceeded according to the process described in the most recent Maker Wiki tutorial (Holt and Yandell 2018). A custom repeat library was created using RepeatModeler v1.0.11 (Smit and Hubley 2008). The transcriptome assembly, genome assembly, and proteins from UniProtKB Swiss-prot (Boutet *et al.* 2007; The Uniprot Consortium 2019) were used as input to MAKER to create initial annotations. Gene models based on these annotations were used to train the following *ab initio* gene predictors: AUGUSTUS v3.3.2 (Stanke and Waack 2003; Stanke *et al.* 2006) and SNAP downloaded 3 June 2019 (Korf 2004). AUGUSTUS was trained using BUSCO (Simão *et al.* 2015) as a wrapper; SNAP was trained without a wrapper. Genemark-ES v4.38 (Lomsadze *et al.* 2005, 2014; Brůna *et al.* 2020) was also trained, though necessarily without the initial models from MAKER. These models were all provided to MAKER for a second round of structural annotation. The gene models based on those annotations were filtered with gFACs v1.1.1 (Caballero and Wegrzyn 2019) and again provided to AUGUSTUS and SNAP. As Genemark-ES does not accept initial gene models, it had no need to be run again. The gene models from the *ab initio* gene predictors were again provided to MAKER for a third and final round of annotation. Functional annotations were added using MAKER accessory scripts, the BLAST+ Suite v2.9.0 (Altschul *et al.* 1990; Camacho *et al.* 2009), and InterProScan v5.45-80.0 (Jones *et al.* 2014; Mitchell *et al.* 2019).

### Demographic history

We inferred the historical demography of *C. melampygus* and its close relative, the giant trevally (*Caranx ignobilis*), by implementing the multiple sequentially Markovian coalescent (MSMC) model (Schiffels and Durbin 2014) to generate estimates of effective population size (*N*_e_) over time. MSMC estimates the rate of coalescent events between two alleles at each locus along an unphased, single diploid genome. We used the self-corrected PacBio reads mapped against the assembly, filtered for contigs > 500 Kb, and applied additional cutoffs to ensure sufficient sequencing depth and quality using MSMC-tools downloaded October 8, 2020 (https://github.com/stschiff/msmc-tools; Mather *et al.* 2020; Schiffels and Wang 2020). We used a draft *de novo* genome for *C. ignobilis* (Glass *et al.* 2021). We ran MSMC v1.1.0 using the following time patterning parameters to estimate 20-time intervals and one free coalescent rate parameter: “1*2 + 16*1 + 1*2”. We then generated 1000 bootstrap estimates using a simulated dataset that randomly pulled, with replacement, 500 Kb long segments and arranged them into 52 segments per “chromosome”. We generated 30 simulated “chromosomes” to construct artificial 780 Mb long genomes, reflecting the estimated size of the *C. melampygus* genome, to determine confidence intervals around *N*_e_ estimates. We used the same MSMC parameters for *C. ignobilis*, except that the 30 simulated “chromosomes” contained 42 segments of length 500 Kb to construct 630 Mb long genomes to reflect the estimated size of the *C. ignobilis* genome (Hardie and Hebert 2004; Gregory 2018). After running MSMC, we converted population sizes and times into number of individuals and years, respectively, using a per site per generation mutation rate (μ = 3.7 e^−8^) from another marine teleost species (Liu *et al.* 2016). For *C. melampygus*, we used a generation time of four, based on the average age of sexual maturity of *C. melampygus* (two) multiplied by two (Nadachowska-Brzyska *et al.* 2016; Mather *et al.* 2020). For *C. ignobilis*, we used a generation time of six, given an average age of three for sexual maturity in this species. The scripts to perform this analysis are available on GitHub (https://github.com/pickettbd/msmc-slurmPipeline) with supporting documentation.

## Results and discussion

### Sequencing

**Figure 2:**
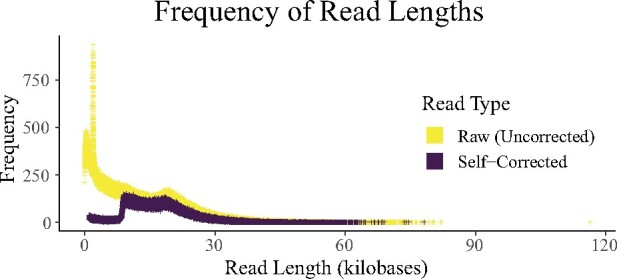
Frequency of pacific biosciences read lengths. The change in read length distribution is demonstrated as reads are corrected. The dramatic shift from raw to corrected reads is evident.

CLR sequencing (PacBio) generated 4.45 M reads with a total of 52.67 Gb, which is ∼67× physical coverage of the genome. The mean and N50 read lengths were 11,834.678 and 19,264 base pairs (bp), respectively. The longest read was 116,429 bp. The read length distribution is plotted in Figure 2. A summary of the results for the sequencing run is available in Table 1. This genome represents the first for the *Caranx* genus and ranks among the highest quality genomes available for Carangoidea in terms of N50 (Zhang *et al.* 2019).

**Table 1:** Sequencing information

Company	Illumina	PacBio
Instrument	Hi-Seq 2500	Sequel I
Mode	Rapid run	NA
Sequencing type	PE	SMRT, CLR
Duration	250 cycles	30 hours
Specimen	1	1
Tissues	Brain, eye, fin, gill, heart, kidney, liver, and muscle	Heart
Molecule	RNA	DNA
Millions of Read (Pair)s	257.5	4.5
Mean read length (bp)	222.6	11,834.7
Read N50 (bp)	249	19,264
Nucleotides (Gb)	114.6	52.7

The results from each type of DNA and RNA sequencing from *C. melampygus*.

PE, paired-end reads; SMRT, single-molecule, real-time sequencing; CLR, continuous long-reads.

RNA-seq from the eight tissues (*i.e.*, brain, eye, fin, gill, heart, kidney, liver, and muscle) generated 257.47 M pairs of reads totaling 114.61 Gb. Across all eight tissues, the mean and N50 read lengths were 222.6 and 249 bp, respectively. The combined results from all eight tissues are represented in Table 1, whereas the results from each tissue are made available in Table 2.

**Table 2:** RNA-sequencing details per tissue

	Millions of read pairs	Mean read length	Read N50	Nucleotides (Gb)
Brain	31.3	219.8	249	13.8
Eye	38.0	219.9	249	16.7
Fin	33.0	219.9	249	14.5
Gill	29.0	225.4	249	13.1
Heart	33.0	228.9	249	15.1
Kidney	32.5	222.5	249	14.5
Liver	30.1	224.6	249	13.5
Muscle	30.6	220.3	249	13.5
All	257.5	222.6	249	114.7

The results of RNA sequencing for each tissue from one *C. melampygus* individual. The eight tissues were spread across two lanes and run on an Illumina Hi-Seq 2500 in Rapid Run mode for 250 cycles to generate paired-end reads. Unless otherwise specified, lengths of nucleotide sequences are measured in bp.

### PacBio CLR error correction

The self-correction strategy reduced the number of reads from 4.45 to 1.77 M and the total number of bases from 52.67 to 29.6 Gb for an approximate physical coverage of 37.8×. The mean and N50 read lengths were changed from 11,835 and 19,264 to 16,769 and 19,027 bp, respectively. The longest read was 78,163 bp. The distribution of read lengths can be viewed in Figure 2.

### Genome assembly

**Figure 3:**
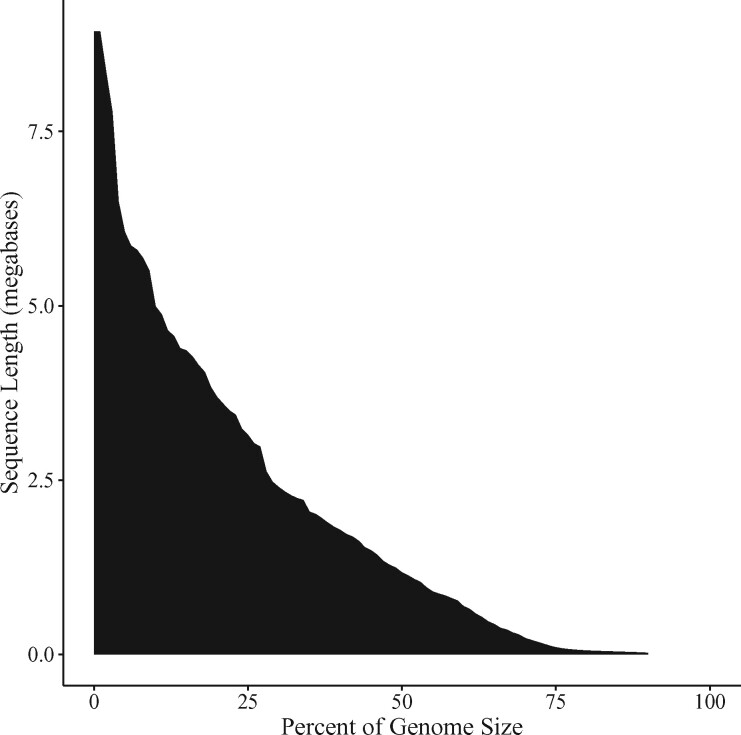
Area under the NG-curve. The NG-curve and the area under it are plotted for the contigs.

The initial assembly from Canu was comprised of 3.6 K contigs with a total assembly size of 711 Mb. The mean contig length, N50, NG50, and maximum contig length were 198.8 Kb, 1.5 Mb, 1.2 Mb, and 8.9 Mb, respectively. The L50 was 120, and the LG50 was 147. The auNG was 1.93 M. Table 3 summarizes the assembly continuity statistics, and the auNG is visualized in Figure 3.

**Table 3:** Assembly and annotation statistics

	Contigs	Transcripts
Continuity statistics		
Sequences	3,577	679,833
Known bases	711.0 Mb	795.8 Mb
Mean length	198,759.67	1,170.5
Max. length	8,932,605	59,179
NG50	1,176,926	—
NG90	24,428	—
LG50	147	—
LG90	3,179	—
auNG	1,927,338	—
Completeness statistics		
Complete BUSCOs	95.6%	93.3%
Complete, single-copy BUSCOs	89.2%	31.5%
Complete, duplicated BUSCOs	6.4%	61.8%
Fragmented BUSCOs	0.6%	3.1%
Missing BUSCOs	3.8%	3.6%
Annotation information		
Repetitive elements	16.9%	—
Protein-coding genes	33,062	—
Functionally annotated genes	23,622	—
tRNAs	2,126	—
Mean CDS length	9,693.1	—
Max. CDS length	191,745	—

Assembly and annotation statistics for the *C. melampygus* genome assembly. Assembly continuity statistics are presented first, followed by completeness statistics and information about the annotation. The completeness statistics are based on a BUSCO analysis using the Actinopterygii set of 3,460 single-copy orthologs. Note that the auNG value is the area under the NG-curve and is unitless. Also note that some continuity statistics values for the transcriptome assembly are meaningless because the size of the transcriptome is not expected to be the size of the genome. Unless otherwise specified, all nucleotide sequences are measured in bp.

The assembly completeness, as assessed with single-copy orthologs, was also evaluated (Table 3). The final set of contigs had 3,480 complete single-copy orthologs (95.6% of 3,640 from the ODB10 Actinopterygii set). Of these 93.3% (3,248) were present in the assembly only once, and 6.7% (232) were present more than once. Twenty-one (0.6%) and 139 (3.8%) single-copy orthologs were fragmented in and missing from the assembly, respectively. Approximately 16.9% of the genome was comprised of repetitive elements (Table 4), which is similar to other Carangoid genomes, for example, *Pseudocaranx georgianus* at 12.8% (Ruigrok *et al.* 2021) and *T.* *ovatus* at 20.3% (Zhang *et al.* 2019).

**Table 4:** Summary of repeats

	Copies	Length (Mb)	Percent (%) of sequence
Interspersed repeats	603,848	89.7	12.6
** **SINE:	16,320	1.9	0.3
** **Penelope	5,005	1.2	0.2
** **LINE	76,167	16.5	2.3
** **LTR	19,399	4.9	0.7
** **DNA transposon	279,202	38.2	5.4
** **Unclassified	207,755	28.2	4.0
Tandem repeats	569,149	22.6	3.2
** **Satellite	1,289	0.4	0.1
** **SSR	518,053	19.6	2.8
** **Low complexity	49,807	2.6	0.4
Rolling-circles	38,561	7.1	1.0
Small RNA	9,592	1.5	0.2
Total	1,221,150	120.2	16.9

Summary of repeat content in the *C. melampygus* genome assembly as reported by RepeatMasker (Smit *et al.* 2021) using the Dfam v3.3 (Storer *et al.* 2021) and RepBase RepeatMasker v20181026 (Jurka 1998; Bao *et al.* 2015) repeat libraries.

### Transcriptome assembly and computational genome annotation

The transcriptome assembly generated by Trinity was comprised of 680 K sequences with a mean sequence length of 1,171 bp. The N50 and L50 were 2.4 Kb and 89 K, respectively. The N90 and L90 were, respectively, 434 bp and 419 K. Of the 3640 single-copy orthologs in the ODB10 Actinopterygii set, 93.3% (3,399) were complete; 33.8% (1,148) of which were present only once in the transcript set. 112 (3.1%) single-copy orthologs were fragmented in the transcript set, 129 (3.6%) were missing. Computational structural and functional annotation using the transcriptome assembly and the MAKER pipeline yielded 33.1 K protein-coding genes, 71.4% of which were assigned putative functions. Of these, 20.9 and 19.9 K have annotated 5′ and 3′ UTRs, respectively. 2.1 K tRNA genes were also identified. A BUSCO analysis of the annotated genes (as extracted from the assembly with an extra 1 Kb from the ends of each gene) yielded 22.4% (814/3640 of Actinopterygii genes from OrthoDB v10) complete single-copy orthologs. Of the total, 20.1% were present only once, and 2.3% were duplicated. Twelve percent were fragmented, and 65.5% were missing from the annotated genes set. The annotations are available in GFF3 format alongside the assembly.

### Population demography

**Figure 4:**
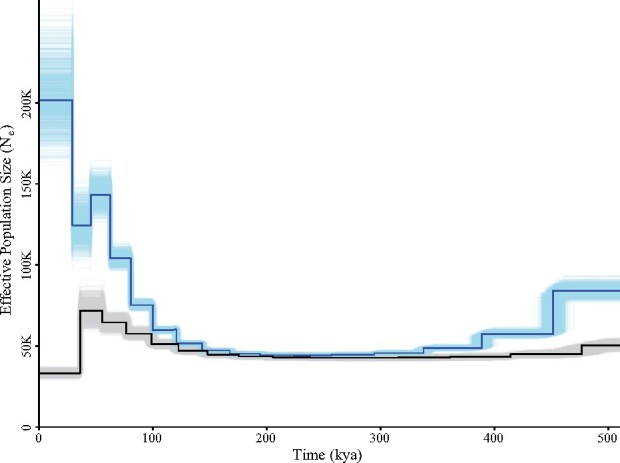
MSMC analysis of demographic history. Inferred demographic history of *C. melampygus* (blue) and *C. ignobilis* (black) over time using MSMC. The darkline represents median effective population size (*N*_e_) estimates. The lightlines indicate 1,000 individual bootstrap replicates.

Results of MSMC modeling indicated a gradual increase in effective population size (*N*_e_) of both *C. melampygus* and *C. ignobilis* beginning around 150 kya, with strong fluctuations in *C. melampygus* population sizes between ∼30 and 75 kya (Figure 4). The increase in *N*_e_ was greater for *C. melampygus* than *C. ignobilis*. Our observations corroborate a previous demographic analysis of both species from Hawai‘i using mitochondrial loci, which also recovered evidence of population expansion compared with *C. ignobilis* (Santos *et al.* 2011). Other demographic components of wild populations (*e.g.*, population structure, nonrandom mating, selection) are also known to affect estimates of coalescent rates (Mazet *et al.* 2016). For example, decreases in sea level have been linked to the isolation of marine populations (Norris and Hull 2012; Cacciapaglia *et al.* 2021), which would lead to demographic changes such as population structure and nonrandom mating. Sea levels decreased globally from the beginning of the Upper Pleistocene (∼129 kya) until the last glacial maximum (∼19–26 kya), with several fluctuations in-between caused by glacial–interglacial cycles (Grant *et al.* 2014). Moreover, ocean circulation patterns were weaker during glacial periods (Rahmstorf 2002), which would limit connectivity between populations of marine fishes such as *C. melampygus* and *C. ignobilis* that disperse primarily via pelagic larval drifting.

Recent evidence suggests *C. melampygus* and *C. ignobilis* individuals are a genetically unique population in Hawai‘i (Glass *et al.* 2021). During the last glacial maximum, exposed limestone bridges linked the Hawaiian Islands of Maui, Lāna‘i, and Moloka‘i and supported reef habitats which became drowned after sea levels began rising (Grigg *et al.* 2002). These limestone reef features may have created increased habitat availability in Hawai‘i during periods of glaciation and supported population expansion. Notably, these species are large-bodied and associated with coastal habitats, including rock and coral reefs, but are not reef-obligate. Overall, some reef fishes exhibit evidence of dramatic declines in population size during glaciation periods (Gaither *et al.* 2009 ), whereas others exhibit evidence of population expansion similar to what is reported here for *C. melampygus* (Delrieu-Trottin *et al.* 2017). An analysis of demographic history for *C. melampygus* individuals from the widespread, Indo-West Pacific population, and individuals of *C. ignobilis* from other identified populations (Glass *et al.* 2021) would allow us to compare population expansion and contractions over time and assess how sea level changes may have affected *C. melampygus* and *C. ignobilis* differently across the Indo-Pacific.

## Conclusion

The assembled genome of *C.* *melampygus* represents the first whole-genome assembly and annotation for the genus *Caranx* and second, after the Atlantic Horse Mackerel (*Trachurus trachurus*; Vertebrate Genomes Project 2020), in the clade Carangini, the most speciose subclade of Carangoidea. The high quality of this reference genome builds on previous carangoid whole-genome datasets and is important for delineating stock structure and demographic history of *C. melampygus*, especially given evidence of a unique genetic lineage in Hawai‘i. The bluefin trevally genome is also a valuable resource for comparative phylogenomic studies of carangoid fishes.

## Data availability

Raw reads have been deposited in the National Center for Biotechnology Information (NCBI) Sequence Read Archive (SRA) under BioProject PRJNA670455. The genome assembly and annotations are associated with the same BioProject and can be found in GenBank under accession JAFELL010000000.

Supplementary material is available at *G3* online.

## Supplementary Material

jkab229_Supplementary_Methods
