# Supplementary material for: De novo genome assembly of the marine teleost, bluefin trevally (Caranx melampygus)
Source: G3 (Bethesda). 2021 Jul 14;11(10):jkab229. doi: 10.1093/g3journal/jkab229 (PMC8473972; doi:10.1093/g3journal/jkab229)
Supplement: jkab229_Supplementary_Methods [file jkab229_supplementary_methods.zip › Supplement/SuppMethods.pdf]

Supplementary Bioinformatics Methods

An overview of the methods used in this study was provided in the main manuscript. Where appropriate, additional details, such as the code for custom scripts and the commands used to run software, are provided here.

Read Error Correction

The self-corrected reads were generated using Canu v1.6 (Koren et al. 2017) with the following command:

```
canu -correct \
    -s ${SETTINGS_FILE} \
    -d ${OUTPUT_DIR_NAME} \
    -p ${OUTPUT_PREFIX} \
    -pacbio-raw \
    ${INPUT_PACBIO_READS[@]}
```

The relevant lines of the setting file are included here:

```
genomeSize=782400000
ovsMethod=sequential
gridEngine=slurm
```

Genome Assembly

The genome assembly step will be described. The RepeatMasker run and the calculation of assembly summary statistics will also be described.

Genome Assembly

The assembly was created with Canu v1.6 (Koren et al. 2017) using the already corrected reads from the “self” correction strategy using the following command:

```
canu -trim-assemble \
    -s ${SETTINGS_FILE} \
    -d ${OUTPUT_DIR_NAME} \
    -p ${OUTPUT_PREFIX} \
    -pacbio-corrected \
    ${INPUT_SELF_CORRECTED_PACBIO_READS_FILE}
```

Repeat Masking

RepeatMasker v4.1.2-p1 (Smit et al. 2021) was run on the contigs to classify the repeat content of the genome. RepeatMasker was built with dependencies on RMBlast v2.11.0 (Camacho et al. 2009; Smit et al. 2021), TRF v4.09.1 (Benson 1999), hmmer v3.3.2 (Wheeler and Eddy 2013), and the h5py (https://www.h5py.org) Python (https://www.python.org) package. Repeat masking was performed against the Dfam v3.3 database (Storer et al. 2021) and the RepeatMasker version of RepBase v20181026 (Bao et al. 2015; Jurka 1998). The command to run RepeatMasker was structured like the following:

```
RepeatMasker \
    -engine "rmblast" \
    -species "Carangidae" \
    -alignments \
    -dir "${OUTPUT_DIR}" \
    -html -source -gff -excln \
    ${CONTIGS_FASTA_FILE}
```

Assembly Statistics

Assembly continuity statistics, e.g., N50 and auN (Li 2020), were calculated with caln50 downloaded April 2020 (https://github.com/lh3/calN50) and a custom Python (https://www.python.org) script. caln50 is run using the following simple command:

```
caln50 \
    -s 0.01 \
    -L ${GENOME_SIZE} \
    ${CONTIGS_OR_SCAFFOLDS_FILE} \
    > ${STATISTICS_FILE}
```

The custom Python script is not efficient, but it does calculate Nx, Lx, NGx, and LGx, as well as a few other interesting points about sequences in a fasta file. This script is too long to realistically represent when embedded in the text; it is available on GitHub at https://github.com/pickettbd/basicAsmStatsCalcInPy.

Assembly completness was assessed using single-copy orthologs with BUSCO v4.0.6 (Simão et al. 2015) and OrthoDB v10 (Kriventseva et al. 2019). The BUSCO config file was the not modified from the default aside from the locations of OrthoDB v10 and the binary executables for BUSCO. It was run based on the following command structure:

```
busco \
    --offline \
    --config ${BUSCO_CONFIG_FILE} \
    --cpu ${THREADS} \
    --in ${CONTIGS_OR_SCAFFOLDS_FASTA} \
    --out_path ${OUTPUT_DIR} \
    --out ${OUTPUT_FILE_PREFIX} \
    --mode genome \
    --lineage actinopterygii \
    --augustus_species zebrafish
```

Transcriptome Assembly

The transcripts were assembled using Trinity v2.6.6 (Grabherr et al. 2011), which depended on Bowtie v2.3.4.3 (Langmead and Salzberg 2012), Jellyfish v2.2.10 (Marcais and Kingsford 2011), salmon v0.12 (Patro et al. 2017), and SAMtools v1.6 (Li et al. 2009):

```
trinity \
    --no_version_check \
    --max_memory ${MEMORY} \
    --CPU ${THREADS} \
    --long_reads ${DUAL_CORRECTED_PACBIO_READS} \
    --seqType fq \
    --left ${RNASEQ_READS_LEFT} \
    --right ${RNASEQ_READS_RIGHT} \
    --SS_lib_type FR \
    --normalize_max_read_cov 50 \
    --normalize_by_read_set \
    --min_contig_length 200 \
    --output ${TRINITY_OUTPUT_DIR}
```

Assembly completeness was assessed using single-copy orthologs with BUSCO v4.0.6 (Simão et al. 2015) and OrthoDB v10 (Kriventseva et al. 2019). The command and config file were a match to how BUSCO was run to assess genome assembly completeness, except that the `--mode` option was `transcriptome` instead of `genome`.

Computational Genome Annotation

The MAKER v3.01.02-beta (Holt and Yandell 2011) pipeline was used to annotate the assembly. With a large enough cluster with MPI support, MAKER runs relatively quickly for each round. The general process was described in prose in the main manuscript, but it can be summarized in outline form here:

I. MAKER round #1
II. *ab initio* gene predictors
    a. AUGUSTUS
    b. GeneMark-ES
    c. SNAP
III. MAKER round #2
IV. *ab initio* gene predictors

a. AUGUSTUS

b. SNAP

V. MAKER round #3

VI. MAKER post-processing & functional annotation

As each round of MAKER was run in a nearly identical fashion, the process will be described once, followed by differences between the rounds. Similarly, AUGUSTUS and SNAP will also be described once.

MAKER Round #1

The command to run MAKER is straight-forward, though may vary slightly depending on the implementation of MPI employed by the cluster. The MAKER documentation says to run MAKER with the `mpiexec` command, but `mpirun` was successful for our setup. Running MAKER from a working directory on an NFS drive will almost certainly result in failure unless MAKER is directed where to do its work in a non-NFS temporary directory. This required some extra attention to job cleanup on our cluster, but it was successful when we pointed MAKER to the local drives on the nodes on which it was run, which were mounted at `/tmp`. When calling MAKER from the directory in which the control files exist, the command to start MAKER looks like this:

```
mpirun maker \
    -cpus ${CPUS} \
    -TMP ${MAKER_TMP_DIR}
```

The truly critical parts are in the MAKER control files. Assuming one has a successfully installed and configured version of MAKER available, default control files can be generated in the working directory by running the following command: `maker -CTL`. No modifications were made to the `maker_evm.ctl` file. The `maker_bopt.ctl` file was left unchanged as well. Note

that `use_rapsearch` was set to `0` and `blast_type` was set to `ncbi+`. The `maker_exe.ctl` file was modified as needed only to set correct paths to the executables for MAKER's dependencies. The following shows the modified or otherwise relevant lines from the `maker_opts.ctl` file:

```
# genome
genome=/path/to/scaffolds.fa
organism_type=eukaryotic

#re-annotation
maker_gff=
est_pass=0
protein_pass=0
rm_pass=0
model_pass=0
pred_pass=0
other_pass=0

# est/rna-seq
est=/path/to/Trinity/transcripts.fa
est_gff=

# protein homology
protein=/path/to/uniprot_sprot.fa
protein_gff=

# repeat masking
model_org=all
rmlib=/path/to/RepeatModeler/results/assembly-db-families.fa
repeat_protein=/path/to/maker-install-dir/data/te_proteins.fa
rm_gff=
softmask=1
```

```
# gene prediction
snaphmm=
gmhmm=
augustus_species=
pred_gff=
model_gff=
run_evm=0
est2genome=1
protein2genome=1
trna=0

# maker behavior
max_dna_len=1000000
min_contig=20000

pred_flank=200
pred_stats=0
AED_threshold=1
min_protein=0
alt_splice=0
always_complete=0
map_forward=0
keep_preds=0

split_hit=10000
min_intron=20
single_exon=0
single_length=250
correct_est_fusion=0
```

Once MAKER has completed, a few MAKER accessory scripts can be run to extract the results from its datastore located at `${PROJECT_DIR}/maker/rnd1/*.datastore`. Additional modifications (shown) can also be employed to make output names more palatable. For sake of demonstration, we assume the master datastore index log file is prefixed with `scaffolds`, and the output base (`-o` option for `fasta_merge`) is `cmel-rnd1` (*C. melampygus* round 1)):

```
cd maker/rnd1/scaffolds.maker.output

fasta_merge \
    -o cmel-rnd1 \
    -d scaffolds_master_datastore_index.log

gff3_merge \
    -n -s \
    -d scaffolds_master_datastore_index.log \
    > cmel-rnd1_noSeq.gff

cd scaffolds_datastore

rename 's/.all.maker./_/' *.fasta # Perl rename, not Linux util
rename 's/fasta/fa/' *.fasta      # Perl rename, not Linux util

awk '{if ($2 == "est2genome") print $0}' \
    cmel-rnd1_noSeq.gff \
    > cmel-rnd1_est2genome.gff

awk '{if ($2 == "protein2genome") print $0}' \
    cmel-rnd1_noSeq.gff \
    > cmel-rnd1_protein2genome.gff

awk '{if ($2 ~ "repeat") print $0}' \
    cmel-rnd1_noSeq.gff \
    > cmel-rnd1_repeats.gff

mv cmel-rnd1*.fa cmel-rnd1*.gff ../..

cd ../../../..
```

ab initio *Gene Prediction*

Three *ab initio* gene prediction programs were run between MAKER rounds 1 and 2. AUGUSTUS and SNAP can take gene models as input, and they are thus able to be run with new models after rounds 1 and 2 of MAKER in preparation for rounds 2 and 3, respectively. GeneMark-ES does not take gene models as input, and it thus needs to be run only one time.

GeneMark-ES

GeneMark-ES required a software key to be run, which can be obtained or re-obtained for free for academic use at any time. GeneMark-ES also requires a configuration file to be run; the default configuration file was used. The following command demonstrates how to run GeneMark-ES:

```
gmes_petap.pl \
    --ES \
    --usr_cfg ${COPY_OF_DEFAULT_CONFIG_FILE} \
    --cores ${THREADS} \
    --sequence ${SCAFFOLDS_ASSEMBLY_FILE}
```

AUGUSTUS

AUGUSTUS training can be handled with BUSCO. Before AUGUSTUS can be trained, configuration files and data from AUGUSTUS and BUSCO will need to be copied to the working directory for this part of the analysis, and the relevant environment variables will need to be reset (which assumes they are properly set in the first place):

```
cp  -r ${AUGUSTUS_CONFIG_PATH} ${PROJECT_DIR}/augustus_config
export AUGUSTUS_CONFIG_PATH=${PROJECT_DIR}/augustus_config

cp  ${BUSCO_CONFIG_FILE} ${PROJECT_DIR}/busco_config.ini
export BUSCO_CONFIG_FILE=${PROJECT_DIR}/busco_config.ini
```

No changes were made to the AUGUSTUS files. The only change made to the BUSCO configuration file was to set `download_path=/path/to/odb10` instead of `./busco_download`. This is assuming OrthoDB v10 has already been downloaded to that location and that the `--offline` flag will be used when running BUSCO. Before training AUGUSTUS, candidate gene regions need to be extracted. This was done with a custom Python script (available at https://github.com/pickettbd/caranx-melampygus_assembly-paper_misc-scripts) and BEDTools v2.28.0 (Quinlan and Hall 2010).

```
python3 generateBedForMrnaExtraction.py \
    maker/rnd1/cmel-rnd1_noSeq.gff \
    scaffolds.fa \
    candidates-rnd1.bed

bedtools getfasta \
    -fi scaffolds.fa \
    -bed candidates-rnd1.bed \
    -fo candidates-rnd1.fa
```

AUGUSTUS was trained by running BUSCO with the same command described in the Assembly Statistics section (i.e., `mode=genome`, `lineage=actinopterygii`, `augustus_species=zebrafish`). To make the AUGUSTUS training parameters generated after running BUSCO available to the next round of MAKER, some post-processing is required:

```
# make dir for final results
mkdir augustus_config/species/cmel

# move to results location
cd "busco-augustus/cmel-rnd1/
    run_actinopterygii_odb10/augustus_output/
    retraining_parameters/BUSCO_cmel-rnd1"

# rename some files and their references to eachother
rename \ # Perl rename, not Linux util
    's/BUSCO_(cmel-rnd1_)/$1/' \
    ./*

sed \ # gnu sed
    -i -r \
    's/BUSCO_(cmel-rnd1_)/\1/' \
    ./cmel-rnd1_parameters.cfg*

# do it again, removing the rnd info
rename \ # Perl rename, not Linux util
    's/(cmel)-rnd1)/$1/' \
    ./*

sed \ # gnu sed
    -i -r \
    's/(cmel)-rnd1/\1/' \
    ./*

# copy the files to final results location
cp -f ./* ../../../../../../augustus_config/species/cmel/
```

```
# move back to main project dir
cd -
```

SNAP

Training with SNAP is much less resource intensive than training AUGUSTUS. Most, if not all, of the commands can reasonably be run “locally” on a login node or other machine. The final output file, `genome.hmm`, is what will be provided to the next round of MAKER. Inspection of the log files was performed after each step. The process of training SNAP can be described by the following commands:

```
mkdir -p snap/rnd1

ln -s \
    ../../maker/rnd1/cmel-rnd1_withSeq.gff \
    snap/rnd1/genome.gff

cd snap/rnd1

maker2zff genome.gff

fathom \
    genome.ann genome.dna \
    -gene-stats \
    > gene-stats.log

fathom \
    genome.ann genome.dna \
    -validate \
    > validate.log

fathom \
    genome.ann genome.dna \
    -categorize 1000 \
    > categorize.log

fathom \
    uni.ann uni.dna \
    -export 1000 -plus \
    > export.log

forge \
    export.ann export.dna \
    > forge.log
```

```
hmm-assembler.pl \
    genome params \
    > genome.hmm
```

MAKER Round #2

The second round of MAKER was run much the same way as the first, with a few modifications. First, the second round was run in a separate directory: `maker/rnd2`. The `run_evm` flag was set to enable MAKER to run EVidenceModeler v1.1.1 (Haas et al. 2008). The control files were copied from the first round and the following changes were made to `maker_opts.ctl`:

```
# est/rna-seq
est=
est_gff=/path/to/project/maker/rnd1/cmel-rnd1_est2genome.gff

# protein homology
protein=
protein_gff=/path/to/project/maker/rnd1/cmel-rnd1_protein2genome.gff

# repeat masking
model_org=
rmlib=
repeat_protein=
rm_gff=/path/to/project/maker/rnd1/cmel-rnd1_repeats.gff

# gene prediction
snaphmm=/path/to/project/snap/rnd1/genome.hmm
gmhmm=/path/to/project/gmes/output/gmhmm.mod
augustus_species=cmel
run_evm=1
est2genome=0
protein2genome=0
```

Additionally, the same accessory scripts, renaming, etc. was performed after this second round of MAKER as with the first round. The only differences being that `rnd1` was replaced with `rnd2` in all the commands and names and the `awk` commands were skipped.

ab initio *Gene Prediction*

Since GeneMark-ES does not take gene models as input, only SNAP and AUGUSTUS could be re-run after MAKER's second round. Before training them, the models from MAKER were filtered using gFACs v1.1.1 (Caballero and Wegrzyn 2019).

gFACs Filtering

In an attempt to improve the quality of gene models being used for this final round of training with AUGUSTUS and SNAP, gFACs was employed to filter out models with single-exon genes, introns shorter than 20bp, etc. The gFACs command and relevant supporting commands (e.g., creating working directories) are shown here:

```
mkdir -p gfacs/rnd2

ln -s \
    ../../maker/rnd2/cmel-rnd2_noSeq.gff \
    gfacs/rnd2/orig_noSeq.gff

ln -s \
    ../../assembly/scaffolds.fa \
    gfacs/rnd2/assembly.fa

awk \
    'BEGIN{x=0;}/^##FASTA/{x=1;}{if(x){print $0;}}' \
    maker/rnd2/cmel-rnd2_withSeq.gff \
    > gfacs/rnd2/orig_onlySeq.gff

cd gfacs/rnd2

gFACs.pl \
    -f "maker_2.31.9_gff" \
    -p  ./output/cmel-rnd2_noSeq \
    --statistics-at-every-step \
    --statistics \
    --rem-monoexonics \
    --min-exon-size 20 \
    --min-intron-size 20 \
    --min-CDS-size 74 \
    --fasta assembly.fa \
    --splice-table \
    --nt-content \
    --canonical-only \
    --rem-genes-without-stop-codon \
    --allowed-inframe-stop-codons 0 \
```

```
    --create-gff3 \
    --get-fasta-with-introns \
    --get-fasta-without-introns \
    --get-protein-fasta \
    --distributions \
    exon_lengths \
    intron_lengths \
    CDS_lengths \
    gene_lengths \
    exon_position \
    exon_position_data \
    intron_position \
    intron_position_data \
    -O ./output \
    orig_noSeq.gff

ln -s \
    cmel-rnd2_noSeq_out.gff3 \
    output/cmel-rnd2_noSeq.gff

cat \
    output/cmel-rnd2_noSeq.gff orig_onlySeq.gff \
    > output/cmel-rnd2_withSeq.gff

cd ../..
```

AUGUSTUS

Training AUGUSTUS after the second round of MAKER in preparation for the third round occurred in the same manner as the first time. The exceptions were that (a) the input GFF3 file came from gFACs instead of directly from MAKER, (b) `augustus_species=cmel` was used instead of `augustus_species=zebrafish`, and (c) the occurrences of `rnd1` in the commands and names were changed to `rnd2`. The commands are replicated (and appropriately modified) again here:

```
python3 generateBedForMrnaExtraction.py \
    gfacs/rnd2/output/cmel-rnd2_noSeq.gff \
    scaffolds.fa \
    candidates-rnd2.bed
```

```
bedtools getfasta \
    -fi scaffolds.fa \
    -bed candidates-rnd2.bed \
    -fo candidates-rnd2.fa
```

AUGUSTUS was trained by running BUSCO with the same command described in the Assembly Statistics section (i.e., `mode=genome` and `lineage=actinopterygii`) except that `augustus_species=cmel` instead of `zebrafish`. To make the AUGUSTUS training parameters generated after running BUSCO available to the next round of MAKER, some post-processing is required:

```
# move to results location
cd "busco-augustus/cmel-rnd2/
    run_actinopterygii_odb10/augustus_output/
    retraining_parameters/BUSCO_cmel-rnd2"

# rename some files and their references to each other
rename \ # Perl rename, not Linux util
    's/BUSCO_(cmel-rnd2_)/$1/' \
    ./*

sed \ # gnu sed
    -i -r \
    's/BUSCO_(cmel-rnd2_)/\1/' \
    ./cmel-rnd1_parameters.cfg*

# do it again, removing the rnd info
rename \ # Perl rename, not Linux util
    's/(cmel)-rnd2)/$1/' \
    ./*

sed \ # gnu sed
    -i -r \
    's/(cmel)-rnd2/\1/' \
    ./*

# copy the files to final results location
cp -f ./* ../../../../../../augustus_config/species/cmel/

# move back to main project dir
cd –
```

SNAP

Training SNAP after the second round of MAKER in preparation for the third round occurred in the same manner as the first time. The exceptions were that (a) the input GFF3 file came from gFACs instead of directly from MAKER, (b) the `maker2zff` command had to be modified, and (c) the occurrences of `rnd1` in the commands and names were changed to `rnd2`. The `maker2zff` script provided by MAKER that was modified is referred to as `maker2zff_v2`. The only change required was to use `exon` instead of `CDS` on line 142. The commands are replicated (and appropriately modified) again here:

```
mkdir -p snap/rnd2

ln -s \
    ../../gfacs/rnd2/output/cmel-rnd2_withSeq.gff \
    snap/rnd2/genome.gff

cd snap/rnd2

maker2zff_v2 -n genome.gff

fathom \
    genome.ann genome.dna \
    -gene-stats \
    > gene-stats.log

fathom \
    genome.ann genome.dna \
    -validate \
    > validate.log

fathom \
    genome.ann genome.dna \
    -categorize 1000 \
    > categorize.log

fathom \
    uni.ann uni.dna \
    -export 1000 -plus \
    > export.log

forge \
    export.ann export.dna \
    > forge.log
```

```
hmm-assembler.pl \
    genome params \
    > genome.hmm
```

MAKER Round #3

The third round of MAKER was run much the same way as the second, with a few modifications. First, the third round was run in a separate directory: `maker/rnd3`. The `trna` flag was used to ensure MAKER ran tRNAscan-SE v1.3.1 (Chan and Lowe 2019). The control files were copied from the second round and the following changes were made to `maker_opts.ctl`:

```
# gene prediction
snaphmm=/path/to/project/snap/rnd2/genome.hmm
trna=1
```

Additionally, the same accessory scripts, renaming, etc. was performed after this third round of MAKER as with the second round. The only difference being `rnd2` replaced with `rnd3` in all the commands and names (the `awk` commands were again skipped).

MAKER Post-processing and Functional Annotation

The structural annotations created by MAKER required some modest post-processing before adding functional annotations. MAKER accessory scripts were used to update sequence names from the long MAKER names to friendlier ones. Other MAKER scripts were used to update the fasta and/or gff3 files with functional annotations found with the BLAST+ Suite v2.9.0 (Camacho et al. 2009; Altschul et al. 1990) and InterProScan v5.45-80.0 (Jones et al. 2014; Mitchell et al. 2019). BLAST was run using the annotated protein sequences as the query and UniProt/Swissprot as the subject database. The following options were used: `-task blastp -max_target_seqs 1 -max_hsps 1 -evalue 1e-6 -outfmt 6`. InterProScan was run using

annotated proteins as input (same as BLAST) with the following options: `-appl pfam -dp -f TSV -goterms -iprlookup -pa -t p`.

```
# create and move to a working dir
mkdir -p maker/post
cd maker/post

# copy the requisite output files
cp ../rnd3/*.gff ../rnd3/*.fa .
cp ../rnd1/cmel-rnd1_{repeats,{est,protein}2genome}.gff .

# remove the rnd info
rename \ # Perl version, not Linux util
    's/-rnd[1-3]//' \
    *.fa *.gff

# map new ids to MAKER names
NUM_SEQS=`grep -Ev '^#' cmel_noSeq.gff \
    | cut -d "\t" -f 9 | tr ';' '\n' \
    | cut -d '=' -f 2 | sort -u | wc -l`

maker_map_ids \
    --initial=1 \
    --prefix=Caranx-melampygus\
    --suffix='-?%' \
    --iterate=1 \
    --justify=${#NUM_SEQS} \
    cmel_withSeq.gff \
    > identifiers_map.tsv

# rename based on new ids
for FASTA in *.fa
do
    cp -f "${FASTA}" "${FASTA%.fa}_renamed.fa"
    map_fasta_ids identifiers_map.tsv "${FASTA%.fa}_renamed.fa"
done

for GFF in *.gff
do
    cp -f "${GFF}" "${GFF%.gff}_renamed.gff"
    map_gff_ids identifiers_map.tsv "${GFF%.gff}_renamed.gff"
done

# prep for functional annotation
cd /path/to/swissprot

makeblastdb \
    -dbtype prot \
    -in uniprot_sprot.fa \
    -input_type fasta \
```

```
    -title uniprot_sprot \
    -hash_index \
    -out uniprot_sprot \
    -logfile uniprot_sprot_makeblastdb.log

cd -

# do the alignment for func. annot.
blastp \
    -task blastp \
    -query proteins_renamed.fa \
    -db /path/to/swissprot/uniprot_sprot \
    -num_threads ${THREADS} \
    -max_target_seqs 1 \
    -max_hsps 1 \
    -evalue 1e-6 \
    -outfmt 6 \
    -out proteins-x-uniprotSprot_fmt6.tsv

# update the fasta and gff files with func. annots.
for FASTA in *_renamed.fa
do
    maker_functional_fasta \
        /path/to/swissprot/unitprot_sprot.fa \
        proteins-x-uniprotSprot_fmt6.tsv \
        ${FASTA} \
        > ${FASTA%.fa}_putative-function.fa
done

for GFF in *_renamed.gff
do
    maker_functional_gff \
        /path/to/swissprot/unitprot_sprot.fa \
        proteins-x-uniprotSprot_fmt6.tsv \
        ${GFF} \
        > ${GFF%.gff}_putative-function.gff
done

# run interproscan for more func. annots.
interproscan.sh \
    -m "standalone" \
    -cpu ${THREADS} \
    -T "${TMP}" \
    -appl "pfam" \
    -dp \
    -f "TSV" \
    -goterms \
    -iprlookup \
    -pa \
    -t "p" \
    -i proteins_renamed.fa\
    -o proteins-interproscan.tsv
```

```
# update the gff files with interproscan results
for GFF in {with,no}Seq_renamed_putative-function.gff
do
    ipr_update_gff \
        ${GFF} \
        proteins-interproscan.tsv \
        > ${GFF%.gff}_domain-added.gff
done

for GFF in {with,no}Seq_renamed.gff
do
    iprscan2gff3 \
        proteins-interproscan.tsv \
        ${GFF} \
        > ${GFF%.gff}_visible-iprscan-domains.gff
done

cd ../..
```

Demographic History

The scripts to perform this analysis are available on GitHub (https://github.com/pickettbd/msmc-slurmPipeline) with supporting documentation.

REFERENCES

Altschul, S. F., W. Gish, W. Miller, E. W. Myers, and D. J. Lipman, 1990 Basic Local Alignment Search Tool. J. Mol. Biol. 215:403-410.

Bao, W., K. K. Kojima, and O. Kohany, 2015 Repbase Update, a database of repetitive elements in eukaryotic genomes. Mobile DNA 6 (1):11.

Benson, G., 1999 Tandem repeats finder: a program to analyze DNA sequences. Nucleic Acids Res. 27:573-580.

Caballero, M., and J. Wegrzyn, 2019 gFACs: Gene Filtering, Analysis, and Conversion to Unify Genome Annotations Across Alignment and Gene Prediction Frameworks. Genomics Proteomics Bioinformatics 17 (3):305-310.

Camacho, C., G. Coulouris, V. Avagyan, N. Ma, J. Papadopoulos *et al.*, 2009 BLAST+: architecture and applications. BMC Bioinform. 10:421.

Chan, P. P., and T. M. Lowe, 2019 tRNAscan-SE: Searching for tRNA Genes in Genomic Sequences. Methods Mol. Biol. 1962:1-14.

Grabherr, M. G., B. J. Haas, M. Yassour, J. Z. Levin, D. A. Thompson *et al.*, 2011 Full-length transcriptome assembly from RNA-Seq data without a reference genome. Nat. Biotechnol. 29 (7):644-652.

Haas, B. J., S. L. Salzberg, W. Zhu, M. Pertea, J. E. Allen *et al.*, 2008 Automated eukaryotic gene structure annotation using EVidenceModeler and the Program to Assemble Spliced Alignments. Genome Biol. 9 (1):R7.

Holt, C., and M. Yandell, 2011 MAKER2: an annotation pipeline and genome-database management tool for second-generation genome projects. BMC Bioinform. 12:491.

Jones, P., D. Binns, H.-Y. Chang, M. Fraser, W. Li *et al.*, 2014 InterProScan 5: genome-scale protein function classification. Bioinformatics 30 (9):1236-1240.

Jurka, J., 1998 Repeats in genomic DNA: mining and meaning. Curr. Opin. Struct. Biol. 8 (3):333-337.

Kim, D., B. Langmead, and S. L. Salzberg, 2015 HISAT: a fast spliced aligner with low memory requirements. Nat. Methods 12 (4):357-360.

Koren, S., B. P. Walenz, K. Berlin, J. R. Miller, N. H. Bergman *et al.*, 2017 Canu: scalable and accurate long-read assembly via adaptive k-mer weighting and repeat separation. Genome Res. 27 (5):722-736.

Kriventseva, E. V., D. Kuznetsov, F. Tegenfeldt, M. Manni, R. Dias *et al.*, 2019 OrthoDB v10: sampling the diversity of animal, plant, fungal, protist, bacterial and viral genomes for evolutionary and functional annotations of orthologs. Nucleic Acids Res. 47 (D1):D807-D811.

Langmead, B., and S. L. Salzberg, 2012 Fast gapped-read alignment with Bowtie 2. Nat. Methods 9 (4):357-359.

Li, H., 2020 auN: a new metric to measure assembly contiguity in *Heng Li's Blog*.

Li, H., B. Handsaker, A. Wysoker, T. Fennell, J. Ruan *et al.*, 2009 The Sequence Alignment/Map format and SAMtools. Bioinformatics 25 (16):2078-2079.

Marcais, G., and C. Kingsford, 2011 A fast, lock-free approach for efficient parallel counting of occurrences of k-mers. Bioinformatics 27 (6):764-770.

Mitchell, A. L., T. K. Attwood, P. C. Babbitt, M. Blum, P. Bork *et al.*, 2019 InterPro in 2019: improving coverage, classification and access to protein sequence annotations. Nucleic Acids Res. 47 (D1):D351-D360.

Patro, R., G. Duggal, M. I. Love, R. A. Irizarry, and C. Kingsford, 2017 Salmon provides fast and bias-aware quantification of transcript expression. Nat. Methods 14 (4):417-419.

Quinlan, A. R., and I. M. Hall, 2010 BEDTools: a flexible suite of utilities for comparing genomic features. Bioinformatics 26 (6):841-842.

Simão, F. A., R. M. Waterhouse, P. Ioannidis, E. V. Kriventseva, and E. M. Zdobnov, 2015 BUSCO: assessing genome assembly and annotation completeness with single-copy orthologs. Bioinformatics 31 (19):3210-3212.

Smit, A. F. A., R. Hubley, and P. Green, 2021 RepeatMasker. https://repeatmasker.org.

Song, L., D. S. Shankar, and L. Florea, 2016 Rascaf: Improving Genome Assembly with RNA Sequencing Data. Plant Genome 9 (3):1-12.

Storer, J., R. Hubley, J. Rosen, T. J. Wheeler, and A. F. Smit, 2021 The Dfam community resource of transposable element families, sequence models, and genome annotations. Mobile DNA 12 (1):2.

Wheeler, T. J., and S. R. Eddy, 2013 nhmmer: DNA homology search with profile HMMs. Bioinformatics 29 (19):2487-2489.
